# Changes in SARS-CoV-2 viral load and titers over time in SARS-CoV-2-infected human corpses

**DOI:** 10.1371/journal.pone.0287068

**Published:** 2024-03-27

**Authors:** Sayaka Nagasawa, Yuichiro Hirata, Sho Miyamoto, Seiya Ozono, Shun Iida, Harutaka Katano, Shigeki Tsuneya, Kei Kira, Susumu Kobayashi, Makoto Nakajima, Hiroyuki Abe, Masako Ikemura, Isao Yamamoto, Kimiko Nakagawa, Kazumi Kubota, Shinji Akitomi, Iwao Hasegawa, Tetsuo Ushiku, Tadaki Suzuki, Hirotaro Iwase, Yohsuke Makino, Hisako Saitoh

**Affiliations:** 1 Department of Legal Medicine, Graduate School of Medicine, Chiba University, Chiba, Japan; 2 Department of Pathology, National Institute of Infectious Diseases, Tokyo, Japan; 3 Department of Forensic Medicine, Graduate School of Medicine, The University of Tokyo, Tokyo, Japan; 4 Department of Pathology, Graduate School of Medicine, The University of Tokyo, Tokyo, Japan; 5 Department of Forensic Medicine, Kanagawa Dental University, Kanagawa, Japan; 6 Public Interest Incorporated Association Nihon Kousei-Kyoukai, Kanagawa, Japan; 7 Department of Healthcare Information Management, The University of Tokyo Hospital, Tokyo, Japan; 8 Japan Medical Association Research Institute, Tokyo, Japan; 9 International Research Institute of Disaster Science, Tohoku University, Miyagi, Japan; 10 Department of Forensic Dentistry, Graduate School of Medicine and Dental Sciences, Tokyo Medical and Dental University, Tokyo, Japan; Cairo University Faculty of Veterinary Medicine, EGYPT

## Abstract

High viral titers of infectious severe acute respiratory syndrome coronavirus 2 (SARS-CoV-2) have been detected in human corpses long after death. However, little is known about the kinetics of infectious SARS-CoV-2 in corpses. In this case series study, we investigated the postmortem kinetics of infectious SARS-CoV-2 in human corpses by collecting nasopharyngeal swab samples at multiple time points from six SARS-CoV-2-infected patients after their death. SARS-CoV-2 RNA was detected by quantitative reverse transcription-polymerase chain reaction from nasopharyngeal swab samples collected from all six deceased patients. A viral culture showed the presence of infectious virus in one deceased patient up to 12 days after death. Notably, this patient had a shorter time from symptom onset to death than the other patients, and autopsy samples showed pathological findings consistent with viral replication in the upper respiratory tract. Therefore, this patient died during the viral shedding phase, and the amount of infectious virus in the corpse did not decrease over time up to the date of autopsy (12 days after death). The findings of this study indicate that the persistence of SARS-CoV-2 in corpses can vary among individuals and may be associated with the stage of the disease at the time of death. These important results complement many previously reported findings on the infectivity of SARS-CoV-2 at postmortem.

## Introduction

The number of deaths from coronavirus disease 2019 (COVID-19) caused by severe acute respiratory syndrome coronavirus 2 (SARS-CoV-2) has exceeded 6.9 million worldwide as of July 2023 and is still increasing [1]. The number of people who handle SARS-CoV-2 infected corpses (e.g., doctors, nurses, funeral service personnel, and autopsy workers) has increased with the increase in the number of deaths. To prevent infection of the handler and the subsequent spread of infection among people, determining whether and how SARS-CoV-2 is transmitted from dead to living bodies is important.

COVID-19 has respiratory and non-respiratory manifestations, and the respiratory burden is the greatest. However, in severe and fatal patients, SARS-CoV-2 is thought to be disseminated throughout the body with systemic symptoms, such as multiple organ failure and shock [2–4]. Autopsies of patients who died of COVID-19 have shown that viral RNA is detected in various organs throughout the body, and that the viruses detected are capable of infection [5–9]. In addition, infectious SARS-CoV-2 can be detected even in corpses long after death, with high viral titers found in the nasopharynx and lungs of corpses up to 17 days after death [9].

The transmission of various pathogens, such as tuberculosis, hepatitis, human immunodeficiency virus, and abnormal prions, from infected human corpses to living individuals, especially those handling corpses, such as autopsy workers, has been reported [10]. There have been no reports on the transmission of SARS-CoV-2 from human corpses to living humans. However, previous studies have shown transmission of this virus from SARS-CoV-2-infected hamster corpses to non-infected live hamsters [11]. Although animals and humans differ in the amount of virus required for infection, transmission from corpses to living people may occur.

A previous study compared the relationship between a decrease in the SARS-CoV-2 viral titer and temperatures at 4°C, 22°C, 37°C, 56°C, and 70°C [12]. This study showed that the viral titer was stable at 4°C for a long time, and that the viral titer decreased over a short time as the temperature increased. These reports suggest that viruses with infectivity continue to exist even after death in bodies normally stored at 4°C. However, a study of temporal changes in the infectious titer using rats showed that the risk of infection from corpses of rats that had been dead for a long time was low [13]. This finding was also observed when rats were refrigerated because the viral titer decreases with time after death, even at 4°C. However, applying animal results directly to humans is difficult because human and animal corpses differ in many aspects, such as the body size and cadaveric condition. In addition, changes in infectivity titers in human corpses over time have not been studied.

In this study, we analyzed nasopharyngeal swab samples of six autopsy patients who died while they were infected with SARS-CoV-2, and examined changes over time in infectious SARS-CoV-2 titers in human corpses.

## Methods

### Ethics

This study was approved by the Biomedical Research Ethics Committees at the Graduate School of Medicine, Chiba University and The University of Tokyo. Written informed consent was obtained from a family member of each case included in the study.

### Cases and sample collection

**Fig 1:**
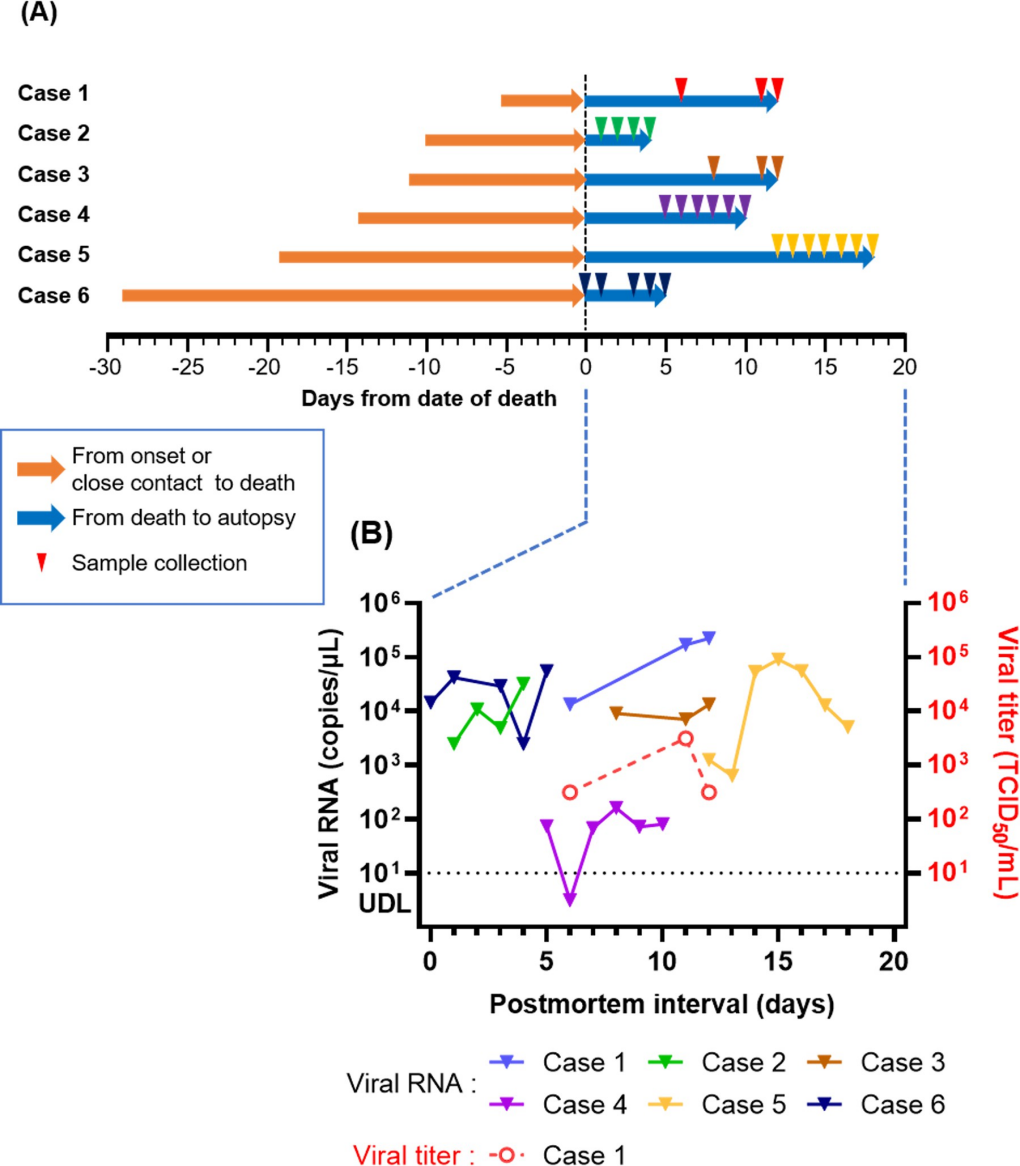
Timeline of cases and changes in the SARS-CoV-2 load or titer in nasopharyngeal swab samples. (A) Time course and sample collection dates for each case with measurement in days from the date of death. Orange arrows: time course from the onset of COVID-19 symptoms (Cases 1, 2, and 4–6) or close contact with a SARS-CoV-2-positive person (Case 3) to death. Blue arrows: time course from death to autopsy. (B) SARS-CoV-2 load (triangles and lines) and titer (red circles and dashed lines, shown only for Case 1) in nasopharyngeal swab samples. The virus could not be isolated from the other five cases. UDL, under the detection limit (< 10 copies/μL).

We initially included bodies transported to the Kanagawa Dental University Forensic Center, which conducts postmortem computed tomographic examinations of corpses with symptoms of fever or suspected COVID-19 before death, from August 2021 to October 2021. Corpses were included in the study if a SARS-CoV-2 antigen test was positive upon arrival at the center and a quantitative reverse transcription-polymerase chain reaction (RT-qPCR) test was positive at the subsequent health center when they were alive. We were able to obtain consent for this study from the bereaved families for six of these cases. Three of these cases (Cases 1, 2, and 3) are the same cases as those in a previously reported study on the amount of infectious virus in the nasopharynx and lungs at autopsy [8]. The patients’ bodies were transferred to the Department of Forensic Medicine, Graduate School of Medicine, The University of Tokyo and stored in a refrigerator at 4°C. The details at the time of the patients’ death and up to the time of transport to our facility are shown in Table 1. Nasopharyngeal swab samples were collected three to seven times before autopsy (Fig 1A). To minimize the effects of sample degradation, the collected swabs were immediately placed in culture medium (Sugiyama-Gen Co., Ltd., Tokyo, Japan) and stored at −80°C [14]. The autopsies were performed by pathologists. Various organ tissue samples (midbrain, pons, medulla oblongata, olfactory bulb, tonsils, upper and lower lobes of the left lung, upper, middle, and lower lobes of the right lung, heart, liver, left and right kidneys, left and right adrenal glands, esophagogastric junction, pylorus, ileum, and genitalia) and body fluids (blood, pericardiac fluid, left and right pleural fluid, cerebrospinal fluid, urine, and ascites) were collected and frozen at −80°C.

**Table 1:** Mortality status of the six cases.

Case No.	Age (years)/sex	Place of death	Month of death and outside temperature (°C) on the date of death (maximum––minimum)	From death to discovery (days)	Days in the morgue (at another facility + at our facility)
1*	37/M	Home (no medical examination)	September (24.5–21.0)	1	12 (6 + 6)
2*	89/F	Hospital	August (28.7–25.4)	0	4 (1 + 3)
3*	73/M	Home (no medical examination)	September (32.0–24.9)	1	12 (8 + 4)
4	26/M	Home (1 day after discharge from the hospital)	September (26.8–22.6)	1	10 (5 + 5)
5	56/M	Home (6 days after discharge from the hospital)	September (27.4–19.9)	0–6	13 (7 + 6)
6	86/F	Hospital	October (18.7–12.9)	0	5 (0 + 5)

*Cases in which viral isolation was performed on lungs collected at autopsy as reported previously (Saitoh et al. [8]).

F, female; M, male.

After obtaining consent from the bereaved families, medical information was obtained from the hospital for hospitalized cases and cases with a history of hospital visits. In addition, information on death at home was also obtained from the police in charge of external surface examinations.

### RT-qPCR

Nucleic acids were extracted from nasopharyngeal swab samples or frozen organ tissues, and body fluids were collected at autopsy using the MagMAX Viral/Pathogen Nucleic Acid Isolation Kit (Thermo Fisher Scientific, Waltham, MA, USA) or the Maxwell RSC Viral Total Nucleic Acid Kit (Promega, Madison, WI, USA). Frozen tissues were cut into small pieces (approximately 10 mg each) and homogenized before extraction. A volume of 1 μL of the obtained nucleic acid solution was used for detecting and quantifying SARS-CoV-2 viral RNA by RT-qPCR [15]. A standard curve was established using data from positive controls (ranging from 10^1^ to 10^6^ copies/reaction). Subsequently, the cycle threshold (Ct) values were converted into viral copy numbers.

### Cell culture and viral isolation

In all RT-qPCR-positive cases, viral isolation was performed using nasopharyngeal swabs by the method described by Yamada et al. [16]. Briefly, VeroE6/TMPRSS2 cells [17] (JCRB1819; Japanese Collection of Research Bioresources Cell Bank) were plated in 96-well flat-bottom plates and then inoculated with the nasopharyngeal swab solution mixed with Dulbecco’s modified Eagle’s medium supplemented with 2% fetal bovine serum and antibiotic-antimycotic solution (Thermo Fisher Scientific, Tokyo, Japan). The culture supernatant was replaced with fresh medium at 1 day post-infection, and the cells were maintained at 37°C with 5% CO_2_. A cytopathic effect was observed on 1 and 5 days post-infection. After 5 days, the supernatant was collected and RT-qPCR using the SARS-CoV-2 direct detection RT-qPCR kit (Takara, Shiga, Japan) was performed to confirm the propagation of SARS-CoV-2. The median tissue culture infectious dose 50% tissue culture infective dose (TCID_50_) in the residual specimens was determined for all viral isolation-positive cases.

### Whole-genome sequencing for SARS-CoV-2

Next-generation whole-genome sequencing of SARS-CoV-2 was performed as described previously on samples from each case (Cases 1–3, 5, and 6) in which a sufficient amount of SARS-CoV-2 (Ct value less than 32, approximately 100 copies per μL of the obtained nucleic acid solution) was detected by RT-qPCR. This sequencing was also performed on culture supernatant containing the isolated virus when available (Case 1) [18]. By following the nCoV-2019 sequencing protocol for Illumina V.5 [19], multiplex RT-qPCR was performed using the primers specified in the protocol, and the obtained PCR products were used to construct a DNA library using the QIAseq FX DNA Library Kit (Qiagen, Hilden, Germany). Genomic sequencing was performed using the MiSeq System (Illumina, San Diego, CA, USA). The obtained consensus viral genome sequences were analyzed with Pangolin COVID-19 Lineage Assigner v.4.3.1 (https://pangolin.cog-uk.io/) to determine the viral lineages. The genomic sequences were registered with the Global Initiative on Sharing All Influenza Data (GISAID; https://www.gisaid.org/).

### Histological examination

In all cases, paraffin-embedded tissue specimens were prepared from tissue samples and used for histological analysis. The prepared tissue sections were stained with hematoxylin and eosin and a rabbit monoclonal antibody against SARS-CoV-2 nucleocapsid (40143-R001; Sino Biological, Beijing, China).

## Results

### Case details

In total 11 corpses were eligible for inclusion of which consent from the bereaved families was obtained for six corpses.

The clinical information of the deceased patients is shown in Table 2. The duration from onset of COVID-19 symptoms (Cases 1, 2, and 4–6) or close contact with a SARS-CoV-2-positive person (Case 3) to death ranged from 5 days (Case 1) to 29 days (Case 6, Fig 1A).

**Table 2:** Clinical information and autopsy findings of each case.

Case no.	Height (cm)/ Weight (kg)	BMI	Past medical history	Autopsy findings
1	162.5/67.1	25.4	Nothing notable	1. DAD and pulmonary congestive edema 2. Previous myocardial infarction
2	150/39.6	17.6	Hypertension, mitral regurgitation, and dementia	1. DAD 2. Papillary thyroid carcinoma
3	163.5/73.6	27.5	Heart failure (details unknown) and diabetes mellitus	1. DAD 2. Diabetes mellitus 3. Clear cell renal cell carcinoma
4	170/93.4	32.3	Atopic dermatitis and migraine	1. DAD and pulmonary congestion
5	161/56.0	21.6	Multiple myeloma	1. Multiple myeloma 2. DAD (organizing phase)
6	146.5/36.0	16.8	Chronic heart failure and Parkinson’s disease	1. Combined pneumonia (invasive pulmonary aspergillosis, COVID-19 pneumonia, and aspiration pneumonia) 2. Age-related brain changes

BMI, body mass index; DAD, diffuse alveolar damage.

### Changes in SARS-CoV-2 copy number and titers in nasopharyngeal swab samples

SARS-CoV-2 viral RNA was detected by RT-qPCR in all nasopharyngeal swab samples collected from Cases 1–3, 5, and 6 up to 18 days after death (Fig 1A). In Case 4, no virus was detected in one sample (6 days after death), but low levels were detected in the other five samples. None of the cases showed a clear decreasing trend in viral RNA load throughout the collection period (Fig 1B). Viral culture showed that infectious virus was isolated from all three samples collected from Case 1 on postmortem days 6, 11, and 12, with titers of approximately 300, 3,000, and 300 TCID_50_/mL, respectively (Fig 1B). Next-generation sequencing showed that sequences from the isolated viruses were identical to those directly from the nasopharyngeal samples (GISAID accession no.: day 6: EPI_ISL_11827882, day 11: EPI_ISL_11827908, and day 12: EPI_ISL_11827869). No infectious virus was isolated from the samples of the other five cases.

### Pathological findings and viral detection at autopsy

The autopsy findings are shown in Table 2. The primary causes of death were COVID-19 (Cases 1–3), multiple myeloma (Case 5), and combined multiple pneumonia (Case 6).

**Fig 2:**
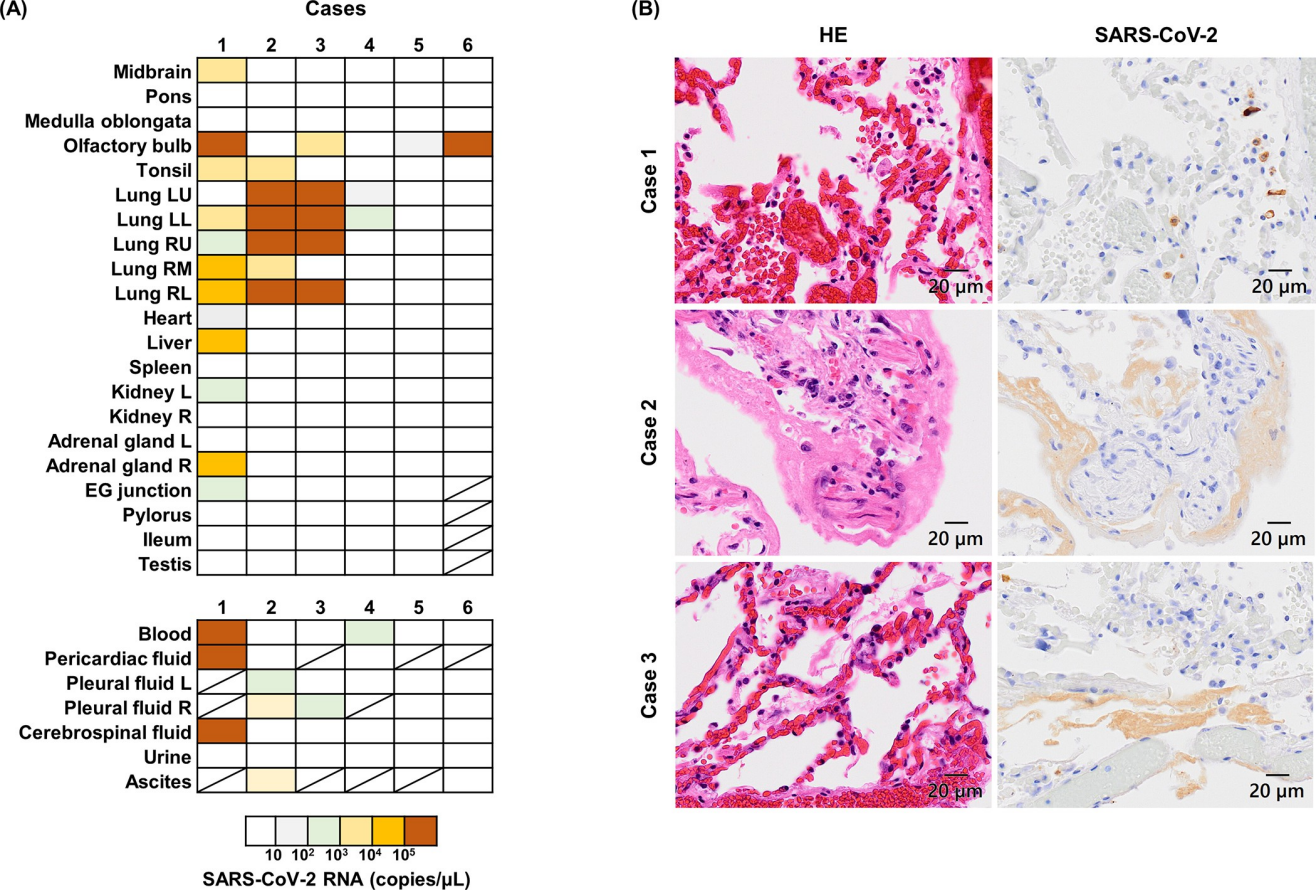
Detection of SARS-CoV-2 at autopsy. (A) Distribution of SARS-CoV-2 RNA detected by RT-qPCR in each case. A crossed box indicates that a sample was not collected. LU, left upper; LL, left lower; RU, right upper; RM, right middle; RL, right lower; EG junction, esophagogastric junction. (B) Representative images of the lungs in each case. The left column shows sections with hematoxylin and eosin staining. The right column shows immunohistochemistry with a rabbit monoclonal antibody that detects SARS-CoV-2 nucleocapsid protein.

RT-qPCR detected high levels of SARS-CoV-2 viral RNA throughout the body in Case 1 and in the lungs in Cases 2 and 3 (Fig 2A). In Cases 4–6, only low levels of the virus were detected in various organs. In Cases 1–3, 5, and 6, next-generation sequencing identified SARS-CoV-2 as the Pango lineage AY.29 (Delta) (GISAID accession no.: Case 1: EPI_ISL_7999834, Case 2: EPI_ISL_7999832, Case 3: EPI_ISL_7999833, Case 5: EPI_ISL_7999838, and Case 6: EPI_ISL_7999835).

A histological evaluation of the lungs showed diffuse alveolar damage in all cases to varying degrees, including lymphocyte infiltration, fibrin deposition in the alveolar space, and hyaline membrane formation (Fig 2B). In Case 1, hyaline membrane formation was limited, and the alveolar space was relatively intact. However, in Cases 2 and 3, fibrin deposition had progressed into the alveolar space, and hyaline membrane formation was extensive. In Case 4, fibrin deposition and hyaline membrane formation were observed in the alveolar space, and the alveolar wall was involved in lymphocytic infiltration. In Case 5, myeloma cell infiltration and were observed in both lungs, but no active inflammation due to SARS-CoV-2 was detected. In Case 6, with focal fibrin deposition was observed. Immunohistochemistry showed a SARS-CoV-2-positive signal in the epithelium and macrophages in Cases 1–3 (Fig 2B). In Case 4, although low levels of the virus were detected in the lower lobe of the left lung (Fig 2A), no obvious positive signal was detected by immunohistochemistry.

## Discussion

In this study, we examined changes over time in the viral load and viral titer of human corpses infected with SARS-CoV-2 from the time of death to autopsy. The viral load was detected in all cases, regardless of the postmortem time, and there was no trend of a decrease in viral load over time. This result suggests that SARS-CoV-2 RNA is stable at postmortem in human and animal species, consistent with previous reports [6,9,13]. However, infectious virus was detected in one of the six cases (Case 1), but only at three time points up to day 12 postmortem. This result is a shorter postmortem time than that previously reported [9]. However, because of the autopsy in this case, we were not able to examine the subsequent period after 12 days postmortem. The high viral titer on day 12 does not rule out the possibility that SARS-CoV-2 remained in the body after this period. Furthermore, our finding is unique in that viral titers did not show a clear decreasing trend at postmortem days 6, 11, and 12, which is different from the temporal changes in viral titers observed in animal cadavers [13]. Although this study is a case series, we believe that our findings provide important information regarding the changes in infectious SARS-CoV-2 titers over time and the risk of infection from human corpses. Our findings suggest that further studies on transmission from corpses are required in the future.

The amount of SARS-CoV-2 replication required for human infection is currently unknown. However, in a human infection experiment conducted in 2021, viral replication was observed in 18 of 34 (approximately 53%) individuals after inoculation with nasal drops containing a low-dose virus at 10 TCID (equivalent to 55 focus-forming units) [20]. The virus was inoculated directly into the nasal cavity in this experiment; therefore, the degree of infection cannot be directly compared with that from a human corpse. However, in a cohabitation experiment of a live hamster in the same cage with a dead hamster infected with SARS-CoV-2, infection from the dead hamster to the living hamster was observed [11]. This infection occurred even when the dead hamster was covered with a wire mesh to prevent direct contact, and the authors speculated that infection was caused by postmortem gas production [11]. Although the amount of virus required for infection and the size and weight of human and hamster corpses are different, gas generation begins in the intestinal tract of human corpses soon after death. Therefore, the virus may be released with the gas and spread into the air when the position of the corpse is changed during transportation or during autopsy. Case 1 in the present study showed a high viral RNA copy number in various organs throughout the body, and Cases 2 and 3 showed SARS-CoV-2 viral RNA in the lungs (Fig 2A). We previously performed viral culture of SARS-CoV-2 in lungs collected at autopsy in these three cases and evaluated viral titers, and found high viral titers of SARS-CoV-2 in all three cases [8]. Although viral titers of other organs have not been evaluated, we consider that SARS-CoV-2 was highly likely to be infectious in other areas in the body. In addition, although this study only included cases with a positive nasopharyngeal swab antigen and PCR test before autopsy, the possibility cannot be excluded that the nasopharynx was negative but the organs were positive for SARS-CoV-2. If the number of cases had not been limited, and if changes over time had been observed in samples other than nasopharyngeal swab specimens, different trends might have been identified. Contact with these specimens from an infected cadaver may increase the risk of infection at autopsy.

The time from symptom onset to death in Case 1 was shorter (5 days) than that in the other five cases. In addition, pathological findings indicated that Case 1 died at a point when the viral load was increasing in the upper respiratory tract [21]. Furthermore, we confirmed the systemic distribution of SARS-CoV-2 RNA in Case 1. Experimental infection shown by a nasopharyngeal swab from humans showed that the viral load peaked at an average of 6.2 days after viral inoculation and tended to decrease thereafter [20]. This study showed that Case 1 died during the viral shedding period, which is the acute phase of infectious symptoms, and the body contained a large amount of infectious SARS-CoV-2 at the time of death. In contrast, there was a long duration between symptom onset and death (at least 10 days) in the other five cases. The pathological findings also indicated that Cases 2–4 were in the phase of hyaline membrane formation, and the viral load was increasing in the lungs. Cases 5 and 6 were in a phase in which the virus was no longer present in the lungs, but fibrosis was progressing [21]. However, no infectious virus was detected in Cases 2–4, indicating that levels of infectious SARS-CoV-2 may have already decreased at the time of death. This possibility indicates that the stage of disease at the time of the patient’s death may be an important factor affecting the postmortem infectivity of SARS-CoV-2.

SARS-CoV-2 is believed to be more stable at lower temperatures [22]. Infectious virus was detected in Case 1, and the corpse was found within half a day after death and stored at 4°C after the confirmation of death. The persistence of SARS-CoV-2 is thought to be greatly affected by the temperature at the time of death, which is affected by the season and the time elapsed between death and the discovery of the corpse. However, unlike dead animal bodies, when a large amount of infectious virus is present in a human corpse at the time of death (e.g., death during the viral shedding period), infectious SARS-CoV-2 remains stable because human corpses are usually stored in a cold environment. Therefore, the risk of infection may persist for a substantial time after death.

## Limitations

A limitation of this study is the small number of SARS-CoV-2-infected cadavers examined. There was a small number of these cadavers because the cases included in this study were SARS-CoV-2-positive cases for which pathological autopsies were performed between August and October 2021. There were few autopsies of SARS-CoV-2-infected corpses performed in Japan during this period. In addition, the corpses sampled in this study were limited to those who were positive for SARS-COV-2 by antigen and PCR before the autopsy and for which autopsy consent was obtained from the bereaved family. Furthermore, because of a lack of staff, some cases were difficult to sample over time, and samples could not be collected on all days, or culture experiments on other organs were not conducted.

## Supporting information

S1 Table PCR raw data. (CSV)
